# Antibiofilm activity of silver nanoparticles against *Candida* sp. isolated from the oral cavity

**DOI:** 10.1590/1414-431X2025e14722

**Published:** 2025-10-17

**Authors:** M.J. Barbaresco, J.S.S.C. da Silva, F.N. de Souza, L.R. Guilherme, P.L.F. Naves

**Affiliations:** 1 Universidade Estadual de Goias, Campus Anápolis de Ciências Exatas e Tecnológicas Henrique Santillo - CCET, Anápolis, GO, Brasil

**Keywords:** Biofilm, Yeast, Colloidal dispersion, Candida krusei, Candida albicans, Antifungal

## Abstract

Candida biofilm is difficult to control due to the poor penetration and unspecificity of antifungal drugs against the microorganisms associated with this structure. Nanoparticles have been investigated for their antimicrobial potential. In this context, the present study evaluated the antibiofilm activity of silver nanoparticle colloids (AgNPs) against candida isolated from the oral cavity. AgNPs were prepared in two different ethanolic syntheses - without and with ammonium hydroxide: AgNP-1 and AgNP-2, respectively. AgNPs were characterized by atomic absorption spectroscopy (AAS), UV-visible (UV-vis) absorption electron spectroscopy, zeta potential, dynamic light scattering (DLS), and transmission electron microscopy (TEM). Subsequently, they were evaluated against biofilm formation by clinical isolates of *Candida albicans* and *Candida krusei* by determining the minimum inhibitory concentration (MIC), minimum fungicidal concentration (MFC), and minimum biofilm formation inhibitory concentration (MBIC_50_). AgNPs inhibited biofilm formation of tested *C. albicans* and *C. krusei* and showed antibiofilm activity at subinhibitory concentrations against all yeasts. AgNP-1 inhibited biofilm formation at concentrations between 7.55 and 60.46 µg/mL and AgNP-2 between 7.71 and 30.80 µg/mL. The characterization of AgNP showed that AgNP-1 and AgNP-2 differ mainly in size and dispersion, with AgNP-2 being monodisperse, indicating that these characteristics could be related to the activity against the formation of *C. albicans* and *C. krusei* biofilm, and the silver nanoparticles may represent innovative and complementary alternatives to the available antifungal arsenal.

## Introduction

Currently, around 150 species of candida have been described, and although infections caused by *C. albicans* constitute the majority of cases, diseases caused by other species are becoming increasingly frequent (1). Candida coexists in the normal microbiota of human mucous membranes and skin. However, due to certain conditions, they increase their affinity for mucous membranes and behave as pathogens in the oral cavity, gastrointestinal, respiratory, and urogenital tracts (2,3).

*Candida* species have several virulence factors, including metabolic adaptability, the ability to alter morphology, expression of surface molecules, secretion of hydrolytic enzymes, and biofilm formation (1). The ability to form biofilms is a relevant factor in *Candida*'s virulence. It protects the fungus from the host's immune responses, limits the penetration of substances through the matrix, and consequently increases resistance to antifungal drugs (4). Species of the genus *Candida* are among the yeasts of greatest medical interest and have the greatest capacity to form biofilms, which make them responsible for around 90% of systemic mycoses and are the fourth most common cause of blood infections in the hospital environment (4).

Biofilm is a structured microbial community interlocked by a protective extracellular matrix and attached to a biotic or abiotic surface. The phenomenon of *Candida* spp. biofilm formation has been observed on surfaces such as mucous membranes, blood vessels, and medical devices that come into direct contact with the patient's body, such as catheters, endotracheal tubes, and implants (4,5).

As resistance to conventional antifungals is inherent in *Candida* spp. biofilms, the treatment of fungal infections associated with this structure, is particularly challenging. The mechanisms of antifungal resistance are varied and complex, but they are attributed to the biofilm's three-dimensional structure, efflux pumps, and persistent cells associated with the biofilm (5).

Silver nanoparticle colloids (AgNPs) are active against yeasts resistant to conventional antifungals, have shown broad-spectrum activity, low resistance induction potential, and have received attention as antibiofilm materials (6,7). The addition of nanomaterials with antibiofilm activity to dental surfaces is another interesting line of research (8). AgNPs can also act synergistically and potentiate active compounds against biofilms, allowing for more efficient use of antimicrobial agents (9).

AgNPs are promising microbial agents in the treatment and control of infectious diseases, as they are active against yeasts due to the release of ions and oxidative stress, damage to the cell membrane and wall, inhibition of enzymatic activity, regulation of gene expression, reduction of ATP, DNA, and protein content, and mitochondrial dysfunction (10,11).

Fungal infections caused by Candida species represent a clinical challenge due to their ability to form biofilm that blocks the action of antifungals and increases the cases of recurrent infections. AgNPs have been studied for their antimicrobial and antifungal potential in combating resistant microorganisms. In this context, it is necessary to investigate the activity of AgNPs against yeasts of the genus *Candida*, particularly the inhibition of biofilm formation and the correlation of inhibitory activity with the characteristics of the synthesized nanoparticles.

## Material and Methods

This quantitative experimental study used a total of seven yeasts isolated from the oral cavity of volunteers (UEG Research Ethics Committee, No. 3.604.978); four were identified as *Candida krusei*, two as *Candida albicans*, and the standard strain *Candida albicans* ATCC 10231 all from the microorganism collection of the Bioassay Laboratory of the Research and Postgraduate Center (LabBio-CPPG) of the State University of Goiás.

### Synthesis and characterization of AgNPs

AgNPs were synthesized through the alcoholic reduction of silver using ethanol as a reducing agent and polyvinylpyrrolidone (PVP) as a stabilizer according to the methodology described by Lee and Oh (12). Briefly, silver nitrate (89.5 mmol/L) and PVP (89.5 mmol/L, Mw 10,000) were dissolved separately in 50 mL of ethanol (*pa*). The two solutions (silver nitrate and PVP) were combined and kept under reflux and constant magnetic stirring for four hours at 80°C, forming a stable AgNP-1. For the synthesis of AgNP-2, 0.3 mL of 28% (v/v) ammonium hydroxide was added to the reaction system when the temperature of 80°C was reached, aiming to increase the conversion of Ag^+^ ions into Ag° atoms, accelerating the ethanol oxidation reaction and influencing the size and distribution of silver nanoparticles.

The synthesized AgNPs were characterized by determining the concentration of silver ions using atomic absorption apectroscopy (AAS) with an AAnalyst 400 spectrophotometer (Perkin Elmer, USA). UV-visible (UV-Vis) spectra were obtained on a 5100 ultraviolet/visible spectrophotometer (Metash, China). The hydrodynamic size (by dynamic light scattering (DLS) and zeta potential were determined using a Zetasizer Pro Blue unit (Malvern Panalytical, UK). Transmission electron microscopy (TEM) and selected area electron diffraction (SAED) studies were performed using a JEOL JEM-2010 200 kV ultra-high-resolution analytical electron microscope (Japan).

The results were organized in the Google Sheets application (Google, USA), which allowed the construction of graphs and statistical analyses in OriginPro 8.5 (Originlab, USA). The images obtained from TEM were analyzed with ImageJ (National Institutes of Health, USA).

### Biofilm formation assay

Biofilm formation was assessed by detecting total biomass using the crystal violet method described by Zayed et al. (13), with some modifications, and by counting the number of yeasts associated with the biofilm. Yeast inocula were obtained by suspending colonies grown in a sterile physiological solution (SPS) and adjusting the turbidity using the 0.5 McFarland scale. Then, 500 µL of the inocula was transferred to tubes containing 4500 µL of bovine heart and brain infusion broth with 2% sucrose (BHIS), and 200 µL of the inocula were aseptically transferred to 96-well polystyrene microplates with a flat bottom (Cralplast, Cral, Brazil), which were incubated at 35.5°C for 48 h.

After incubation, the microplates were processed by removing the broth with total growth, washing the wells, fixing the biofilms with 96° ethanol, and staining with 0.1% crystal violet. The absorbance (620 nm) of the adhered biofilms was then read on a microplate spectrophotometer (Multiskan FC, Thermo Scientific, USA). The average absorbance value obtained in the non-inoculated control wells was used as a reference for classifying biofilm formation as strong, moderate, weak, or non-forming. All the yeasts were tested in independent triplicates totaling 24 wells, and the results obtained were organized as means and standard deviations.

The quantification of viable yeasts associated with the biofilms was determined by incubating 100 µL of the yeast inocula in Eppendorf microtubes with 900 µL of BHIS broth at 35.5°C for 48 h. The microtubes were then processed by aseptically removing the grown broth after 48 h of incubation, washing 3 times in SPS, and detaching the adhered cells with sonication for 5 min in a 40 KHz ultrasonic bath (Ultronique Q3. 8/40A, Brazil) for 5 min at room temperature. The solution was then homogenized in a vortex for 30 s, diluted, and plated so that the number of yeasts associated with the mature biofilm could be counted. All the yeasts were tested in independent triplicates, and the results obtained were organized as means and standard deviations (13).

### Antifungal activity of AgNPs

The yeasts were subjected to the broth microdilution test to determine the minimum inhibitory concentration (MIC) and the minimum fungicidal concentration (MFC) of the nanoparticles, adapting the method recommended by the Clinical and Laboratory Standard Institute for antimicrobial susceptibility tests (14). AgNPs were diluted in round-bottomed microplates (Olen, China) at 1548 to 12.10 μg/mL for AgNP-1 and 1580 to 12.34 μg/mL for AgNP-2.

The yeast inocula were prepared as previously described for the biofilm formation assay by dissolving typically isolated colonies in SPS. The density of the inocula was adjusted using the 0.5 McFarland scale. After adjustment, a 1:10 dilution was made in BHIS broth, and then 20 µL of inocula were transferred to the wells of microplates.

The MIC was determined by visually reading the lowest concentration of the extract capable of inhibiting yeast growth after incubation at 35.5°C for 48 h. MFC was defined as the lowest concentration of the extracts capable of inhibiting the recovery of viable yeasts after transferring 100 µL of BHIS broth from the wells without detectable turbidity to plates containing CPS agar (Biomérieux, Brazil) after incubation at 35.5°C for 48 h. All the tests were carried out in independent triplicates, and the MIC and MFC values were obtained.

### Activity of AgNPs against biofilm formation

The activity of AgNPs against yeast biofilm formation was evaluated as previously described in a total biofilm biomass assay (13). The concentrations of 967.50 to 7.55 μg/mL of AgNP-1 and 987.50 to 7.71 μg/mL of AgNP-2 were tested in flat-bottomed microplates (Cralplast, Cral), and the absorbance of the biofilms formed in the presence of the nanoparticle concentrations were compared with untreated controls. The lowest concentrations that inhibited biofilm formation by at least 50% were considered the minimum biofilm formation inhibitory concentration (MBIC_50_) (15).

## Results

### Characterization of AgNPs

**Figure 1:**
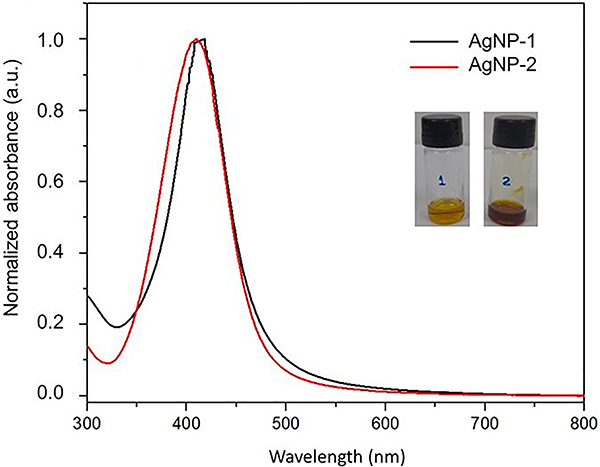
UV-visible absorbance spectrum of silver nanoparticles without ammonium hydroxide (AgNP-1) and silver nanoparticles with ammonium hydroxide (AgNP-2).

The tests for the characterization of AgNPs demonstrated that the concentration of silver ions was 3.870 μg/mL for AgNP-1 and 3.950 μg/mL for AgNP-2. The strong plasmonic bands centered at 418 nm for AgNP-1 and 407 nm for AgNP-2, observed by UV-vis, suggest that the silver nanoparticles are spherical and confirm the yellow coloration (Figure 1).

**Figure 2:**
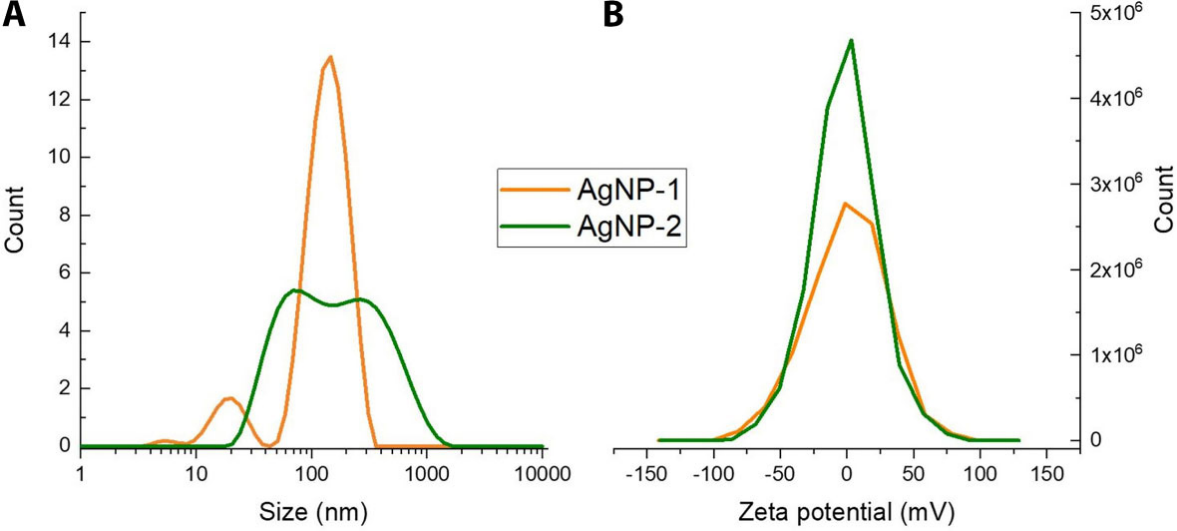
**A**, Hydrodynamic diameter (nm) and **B**, zeta potential (mV) of silver nanoparticle without ammonium hydroxide (AgNP-1) and silver nanoparticle with ammonium hydroxide (AgNP-2).

Figure 2A presents the zeta potential values obtained for AgNPs. The values for determining the zeta potential were close to 0 mV (-0.201 mV for AgNP-1 and -1.784 mV for AgNP-2). These suggest that the colloidal stability of the samples occurs mainly through steric hindrance (steric repulsion) caused by the presence of PVP, which acts as a stabilizer. In this case, colloidal stability occurred due to the PVP layer formed in the ethanolic medium.

The size distribution detected by DLS (Figure 2B) demonstrated that AgNP-1 had a size of 154.2 nm, AgNP-2 had a size of 108.3 nm, and that aggregates could surround the AgNPs. The dispersion indices of the hydrodynamic diameters (DI_H_) were 0.32 and 0.45 nm for AgNP-1 and AgNP-2, respectively. These results demonstrated that the hydrodynamic diameters were not homogeneous.

**Figure 3:**
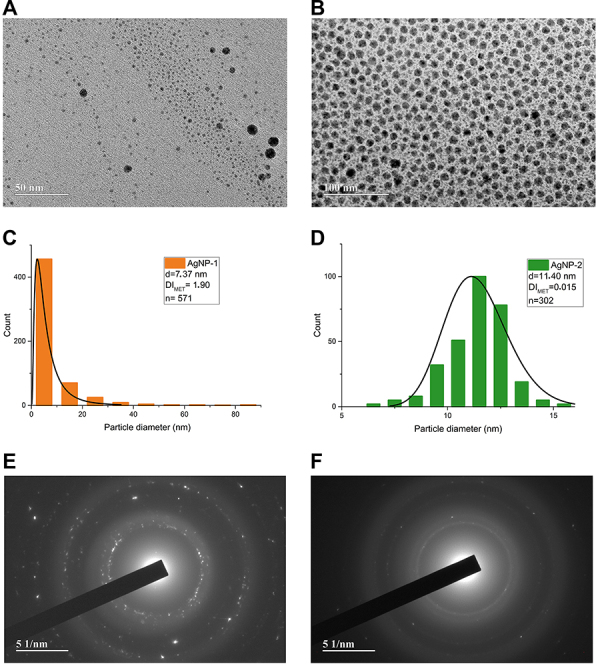
Transmission electron micrograph (TEM) of (**A**) silver nanoparticle without ammonium hydroxide (AgNP-1) and (**B**) silver nanoparticle with ammonium hydroxide (AgNP-2); scale bars 50 and 100 μm. Size distribution histogram of nanoparticles (**C**) AgNP-1 and (**D**) AgNP-2. Selected area electron diffraction (SAED) of AgNP-1 (**E**) and AgNP-2 (**F**); scale bar 51 nm.

TEM analysis showed a size of 7.37 nm for AgNP-1 and 11.40 nm for AgNP-2 (Figure 3A and B). Therefore, spherical AgNPs were obtained. Based on the size distribution histograms (Figure 3C and D), the dispersion index (DI_TEM_) was calculated as 1.90 for AgNP-1, indicating pronounced polydispersity, while 0.015 for AgNP-2 suggested monodispersity. AgNPs with monodispersity have a single and primary population size, as occurs with AgNP-2, and are therefore homogeneous.

Through SAED analyses (Figure 3E and F), the face-centered cubic crystalline phases of the nanoparticles were determined, with no mixture of crystalline phases confirmed by the diffraction rings of the crystalline planes. Crystallographic planes 111, 200, 220, 222, and 422 were found for AgNP-1 and crystallographic planes 200, 321, 422, and 433 were found for AgNP-2 (according to ICSD crystallographic chart No. 00-004-0783).

### Biofilm formation

**Figure 4:**
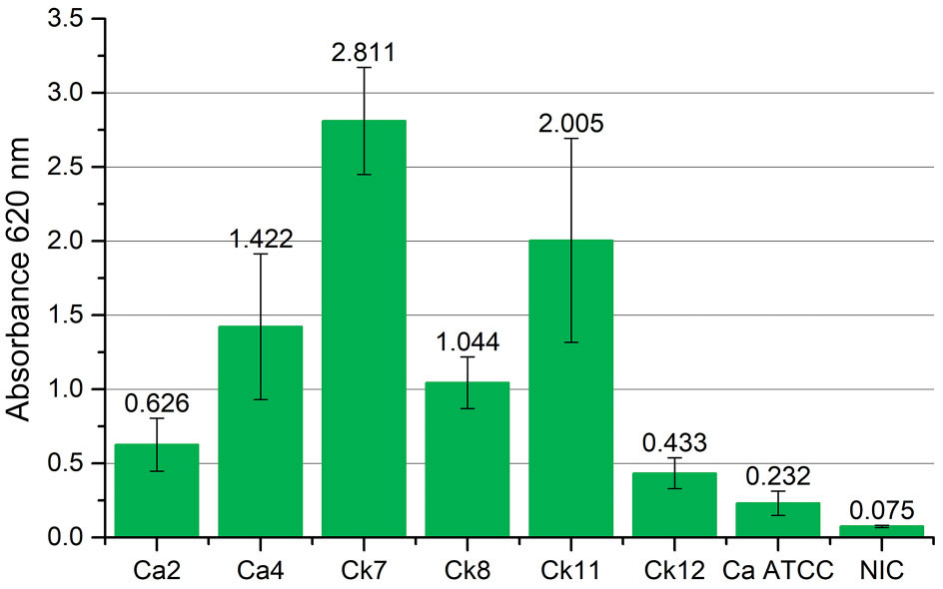
Absorbance (620 nm) readings of yeast biofilms detected by the crystal violet method. Ca: *C. albicans*; Ck: *C. krusei*; ATCC: American Type Culture Collection; NIC: non-inoculated control.

All yeasts formed biofilm under the test conditions. The yeasts *C. krusei* Ck7, *C. krusei* Ck11, *C. albicans* Ca4, and *C. krusei* Ck8 obtained the highest absorbance readings and were classified as strong producers. *C. albicans* Ca2 and *C. krusei* Ck12 were classified as moderate producers, and *C. albicans* ATCC 10231 was identified as a weak producer (Figure 4).

The isolates with the highest counts of yeasts associated with biofilms were *C. krusei* Ck8 (2.48×10^6^ cfu/mL), *C. krusei* Ck7 (2.39×10^6^ cfu/mL), *C. krusei* Ck11 (9.37×10^5^ cfu/mL), *C. albicans* Ca2 (4.70×10^5^ cfu/mL), and *C. krusei* Ck12 (3.95×10^5^ cfu/mL). On the other hand, the yeasts with the lowest results were *C. albicans* Ca4 (8.90×10^4^ cfu/mL) and *C. albicans* ATCC 10231 (2.43×10^4^ cfu/mL). The correlation between the total biomass detected by the crystal violet method and the count of viable cells associated with the biofilm was observed for all the yeasts except for *C. albicans* Ca4.

### Antifungal activity of AgNPs

The results of the antifungal activity of the nanoparticles are shown in Table 1. The nanoparticles inhibited all the yeasts. *C. krusei* was the most sensitive, inhibited by AgNP-1 with MIC=15.11 µg/mL and AgNP-2 with MIC=15.40 µg/mL, while *C. albicans* were most resistant, with MIC=60.46 µg/mL for AgNP-1 and MIC=61.70 µg/mL for AgNP-2.

**Table 1:** Antimicrobial activity of AgNPs (µg/mL) against *C. albicans* and *C. krusei*.

Yeasts	AgNP-1		AgNP-2	
	MIC	MFC	MIC	MFC
*C. albicans* Ca2	60.46	60.46	61.70	61.70
*C. albicans* Ca4	60.46	120.93	61.70	61.70
*C. krusei* Ck7	15.11	120.93	15.40	123.43
*C. krusei* Ck8	15.11	120.93	15.40	123.43
*C. krusei* Ck11	15.11	120.93	15.40	123.43
*C. krusei* Ck12	15.11	120.93	15.40	123.43
*C. albicans* ATCC 10231	60.46	60.46	61.70	61.70

MIC: minimum inhibitory concentration; MFC: minimum fungicidal concentration; AgNP-1: silver nanoparticle without ammonium hydroxide; AgNP-2: silver nanoparticle with ammonium hydroxide.

Regarding fungicidal activity, both nanoparticles (AgNP-1 and AgNP-2) required concentrations 8× higher than the MICs against *C. krusei*. For *C. albicans,* the MFC values were identical to the MICs for most yeasts and double for *C. albicans* Ca4.

### Antibiofilm activity of AgNPs

**Figure 5:**
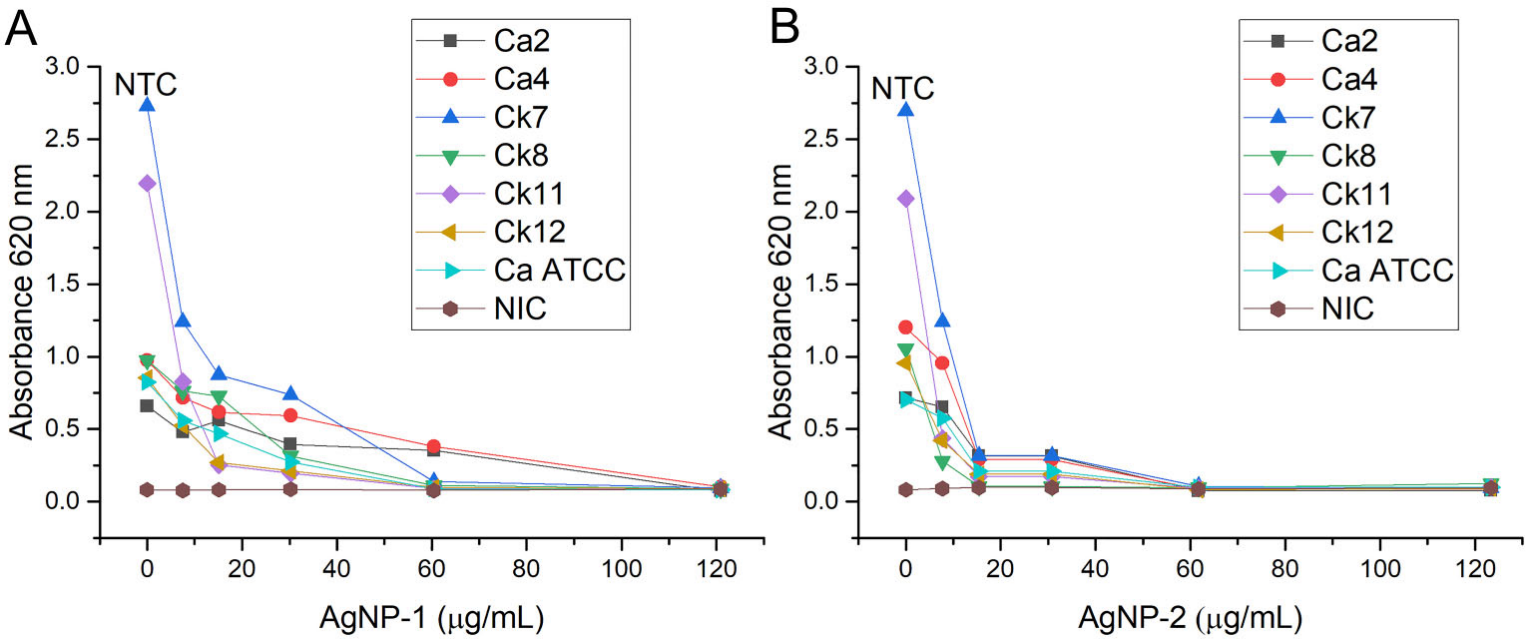
Absorbance (620 nm) readings of yeast biofilms in the presence of silver nanoparticle without ammonium hydroxide (AgNP-1) (**A**) and with ammonium hydroxide (AgNP-2) (**B**) at different concentrations. NTC: Non-treated control; Ca: *C. albicans*; Ck: *C. krusei*; ATCC: American Type Culture Collection; NIC: Non-inoculated control.

Figure 5A and B show the impact of AgNPs concentrations on yeast biofilm formation. The inhibitory activity of the nanoparticles on biofilm formation was concentration-dependent. The highest activity of AgNP-1 (Figure 5A) was detected at 60.46 µg/mL, except for *C. albicans* Ca2 and *C. albicans* Ca4. AgNP-2 (Figure 5B) showed the highest activity at 61.70 µg/mL against all yeasts, but from 15.40 µg/mL, it inhibited biofilm formation.

The inhibition of biofilm formation by AgNP-1 ranged from 7.55 to 60.46 µg/mL. The most sensitive yeasts were *C. krusei* Ck7 and Ck11, with MBIC_50_=7.55 µg/mL. *C. albicans* Ca2 and Ca4 were the most resistant, with MBIC_50_=60.46 µg/mL. The antibiofilm activity of AgNP-2 ranged from 7.71 to 30.80 µg/mL. *C. krusei* was the most sensitive with MBIC_50_=7.71 µg/mL, and *C. albicans* Ca4 was the most resistant with MBIC_50_=30.80 µg/mL (Table 2).

**Table 2:** Antibiofilm activity of AgNPs (µg/mL) against *C. albicans* and *C. krusei*.

Yeasts	AgNP-1			AgNP-2		
	MBIC_50_	MIC	MBIC_50_/MIC (ratio)	MBIC_50_	MIC	MBIC_50_/MIC (ratio)
*C. albicans* Ca2	60.46	60.46	1.0	15.40	61.70	0.25
*C. albicans* Ca4	60.46	60.46	1.0	30.80	61.70	0.5
*C. krusei* Ck7	7.55	15.11	0.5	7.71	15.40	0.5
*C. krusei* Ck8	30.23	15.11	2.0	7.71	15.40	0.5
*C. krusei* Ck11	7.55	15.11	0.5	7.71	15.40	0.5
*C. krusei* Ck12	15.11	15.11	1.1	7.71	15.40	0.5
*C. albicans* ATCC 10231	30.23	60.46	0.5	15.40	61.70	0.25

AgNP-1: silver nanoparticle without ammonium hydroxide; AgNP-2: silver nanoparticle with ammonium hydroxide; MIC: minimum inhibitory concentration; MBIC_50_: minimum biofilm formation inhibitory concentration.

Both nanoparticles (AgNP-1 and AgNP-2) inhibited biofilm formation by the yeasts analyzed. Although the concentrations of silver ions were similar between the two nanoparticles, factors such as the particle sizes, the size distribution of the agglomerates, and the hydrodynamic radius may have influenced the results.

However, AgNP-1 showed yeast-dependent relationships between MBIC_50_ and MIC. While for the yeasts Ck7, Ck11, and Ca ATCC 10231 biofilm formation was inhibited by subinhibitory concentrations (1/2 MIC), the same effect was not observed against *C. krusei* Ck8, as antibiofilm activity was observed at 2× MIC.

AgNP-2 showed a more homogeneous MBIC_50_/MIC ratio among the yeasts, as biofilm formation was inhibited by 1/2 MIC for Ca4, Ck7, Ck8, Ck11, and Ck12, and by 1/4 MIC for Ca2 and Ca ATCC 10231.

Although the inhibitory activity of both nanoparticles was more intense against *C. krusei* than *C. albicans*, the inhibition of biofilm formation by AgNP-2 was more homogeneous among the yeasts tested, which can be explained by its monodispersion of 0.15 per ID_MET_.

## Discussion

### Characterization of AgNPs

Our results indicated that the nanoparticles were spherical, similar in size, and with almost zero surface charges but with different dispersivity indices (Figure 3A and B). The data obtained indicated that AgNP-1 is polydisperse. AgNP-2, synthesized in an ammonium hydroxide reaction medium, is monodisperse, i.e., it presents a homogeneous size distribution (Figure 3C and D). It is known that interactions between AgNPs and microorganisms depend on their physicochemical characteristics, such as hydrodynamic size, surface charge, aggregation (DI_TEM_), and availability of ionic silver release rate (16).

### Biofilm formation

As shown in Figure 4, all colonies formed biofilm under the test conditions. It is known that the total biomass detected by crystal violet is the amount of exopolysaccharides present in the microbial biofilm. Biofilms comprise a population of microbial cells that are adhered to each other and to solid surfaces but are embedded in the self-produced viscous extracellular matrix (17).

Previously, *C. albicans* ATCC 10231 was identified as a strong biofilm producer (18). In another study, the same yeast showed absorbance of 0.175 and viable cell counts of 2.30×10^8^ cfu/mL (19). However, the same strain had the lowest biofilm formation in our study compared to the yeasts isolated from the oral cavity. This difference between the results may be due to the different methodological conditions used in the studies.

The biofilm formation profile is a determining factor in the prognosis of candida infections. This ability may be related to increased mortality, impacting yeast resistance. Yeast infection processes associated with biofilms have a mortality rate almost twice as high as planktonic infections (20).

Candida biofilms are formed by a mixture of extracellular material, yeast cells, and hyphae, which are essential for their development. The transition from yeast to hyphae contributes to the overall virulence of Candida cells and may constitute a significant target for developing antifungal drugs (21).

The ability of Candida yeasts to proliferate and establish biofilms is also influenced by their interaction with host homeostasis and variations, such as mucosal pH changes or nutritional alterations, and by the state of the host immune system (22).

### Antifungal activity of AgNPs

The antifungal activity of the nanoparticles did not differ significantly between AgNP-1 and AgNP-2. However, when we analyzed the difference between the inhibitory and fungicidal concentrations, we found that, for *C. krusei*, the concentrations needed to reach the MFC were eight times higher than the MIC. The same phenomenon was not observed for *C. albicans* (Table 1).

A possible explanation for the fungicidal effect may be related to the spherical shape and average diameter of the AgNPs synthesized in our study (Figure 3A and B). In a previous work, spherical silver nanoparticles with diameters of 2.7 to 12.2 nm were active against planktonic and biofilm-associated *C. albicans* and *C. dubliniensis* with MICs of 7.8 to 15.6 μg/mL and MFCs of 31.2 to 62.5 μg/mL (23). AgNPs showed significant antifungal activity against *C. albicans* with MIC from 62 to 250 μg/mL and MFC from 125 to 500 μg/mL (24), similar to our results.

The fungistatic and fungicidal action of AgNPs is dependent on their concentration (25,26). Nevertheless, the antimicrobial efficiency of nanoparticles also depends on the synthesis methods and the size and shape of the nanoparticles formed (27). AgNPs prepared by different methods show different levels of antifungal activity depending on their size, morphology, and surface modification (28). In addition, the release rate of metallic nanoparticles is related to their chemical composition, structure, metallic core, surface coating, and environmental conditions, such as pH and ionic strength (29).

Metal nanoparticles have a high surface-to-volume ratio, which guides their ability to interact with the membrane of microorganisms and exert their action. The two most commonly accepted theories are death by direct contact due to damage to the cell membrane and protein deactivation caused by the release of Ag^+^ ions (28).

The antifungal activity of silver nanoparticles is based on the fact that they produce reactive oxygen species (ROS) that act on the cell wall and interrupt DNA replication (26,30). In addition, they may be associated with the induction of apoptosis in *C. albicans* cells due to increased oxidative stress and mitochondrial dysfunction (31). A previous study showed that, in the presence of AgNPs, even at a subinhibitory concentration (5 µg/mL), ROS increased by around 73% in yeasts (29).

### Antibiofilm activities of AgNPs

The AgNPs analyzed in our study showed differences in their biofilm inhibitory activities (Table 2). While AgNP-1 inhibited biofilm formation at concentrations similar to the inhibitory concentrations (MIC), AgNP-2 showed MBIC_50_ lower than the inhibitory concentrations, which may indicate a better performance in inhibiting biofilm formation, even at sub-inhibitory concentrations. This observation may be related to the monodispersity of AgNP-2 (Figure 3B and D).

Candida biofilms are notoriously difficult to treat due to their multifactorial resistance, requiring higher concentrations of antifungal drugs compared to the treatment of planktonic cells (6). *Candida krusei* belongs to the group of etiological agents of candidiasis. Although it is not isolated as frequently as other Candida species, its infection is relevant in the clinical environment due to its intrinsic resistance to fluconazole (32). AgNPs disrupt biofilm integrity by interacting with the extracellular polymeric matrix, DNA, lipids, and proteins of biofilms. Such interactions depend on colloidal forces and dynamic biophysical-chemical interactions (33).

The cell wall of *C. albicans* is a dynamic structure modulated to generate elliptical and tubular shapes that define a wide range of morphologies in which this fungus can grow (34). Its chemical composition, although not fully clarified, is composed of an amorphous inner skeletal layer of β(1,3)- and β(1,6)-glucan and chitin and another outer fibrillary layer, which is believed to be dominated by highly mannosylated cell wall proteins of sizes that can vary between 40-60 nm (35).

The morphological changes in the surface of fungal cells may be due to cell wall and membrane damage. The effects exerted by AgNPs on the cell wall and cell membrane may alter the physical state and, therefore, the fluidity of the membrane. The depletion of ergosterol after AgNPs treatment is also thought to be responsible for the sensitivity of fungal cells, the integrity of the membrane, and its changes in the cellular microenvironment (26).

**Figure 6:**
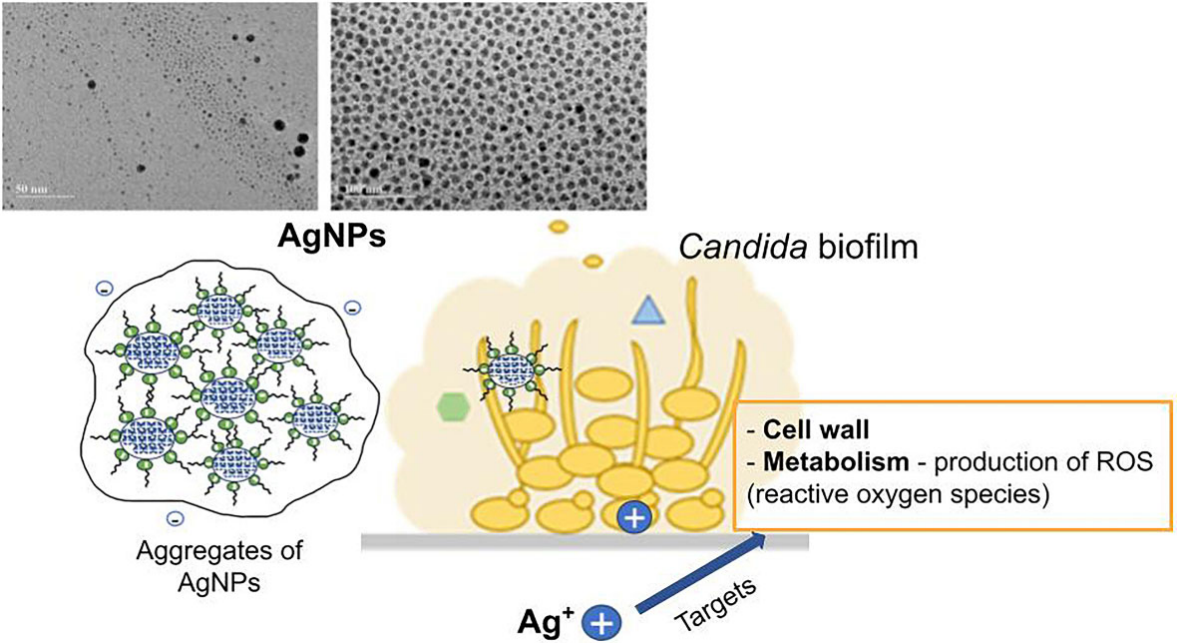
Schematic representation of the action of silver nanoparticles (AgNPs) on *Candida* biofilm. Ag^+^: silver ions

The mechanisms by which AgNPs interact with biofilms determine their activity against the microorganisms associated with this structure. AgNPs cause oxidative stress, protein dysfunction, membrane rupture, and DNA damage, leading to microbial death; they can also alter cell adhesion to prevent biofilm formation (36). In addition, AgNPs exert an inhibitory effect against fungal biofilms by damaging the cell wall, mainly through distortion and rupture of the outer surface of the fungal cell wall. This distortion and rupture cause an increase in ROS and the production of hydroxyl radicals, which also contributes to damage the cell membrane (37). Recent studies suggest that the production and delivery process of the extracellular matrix provides a useful therapeutic target for biofilms of various fungal species (Figure 6) (38).

The physicochemical properties of AgNPs are considered critical factors for their biological applications (11). The activity of AgNPs against yeasts was verified in a previous study, in which AgNPs with sizes ranging from 43.4 to 120.6 nm, a negative charge, and a surface coating showed antifungal activity with MICs ranging from 1.25 to 40 µM against clinical isolates of *Candida albicans* and *Candida krusei*, among other yeasts (39).

Smaller nanoparticles tend to have higher microbicidal effects due to the greater surface area in contact with the microorganism (30). The small size of AgNPs plays a crucial role in their more effective penetration into the cell, influencing the selective permeability of the membrane. Once in contact with the intracellular environment, AgNPs act to reduce certain metabolic functions, slow down vital activities, and lead to cell damage (6).

The interactions between AgNPs and the biofilm can be described by the sequential mechanisms of transport of the nanoparticles to the biofilm-fluid interface, attachment to the biofilm surface, and migration into the biofilm (40).

The type of synthesis, the size and charge of the nanoparticles formed, and the specific interactions of the candida species that make up the biofilm interfere with the interactions with yeast biofilms. The AgNP-induced alteration observed in cellular targets can potentially affect drug resistance, pathogenesis, and virulence of fungal pathogens (26).

The size of the AgNPs mainly controls the initial penetration step into the extracellular polymeric substance (EPS) of the biofilm. At the same time, the surface properties of the AgNPs, such as charge and functional groups, affect the interactions with components of the EPS matrix. When AgNPs come into contact with an environment containing organic molecules, a corona-like coating is formed on the surface of the AgNPs, and the nature of this corona influences AgNP-biofilm interactions (40).

## Conclusion

The results of this study highlight the role of size homogeneity in colloidal dispersions and nanoparticle synthesis in optimizing antimicrobial performance. Incorporating ammonium hydroxide in the synthesis of AgNP-2 improved nanoparticle size homogeneity and enhanced biofilm inhibition, highlighting the importance of designing tailored nanomaterials.

Future studies should explore *in vivo* applications, long-term toxicity, and synergistic effects with conventional antifungals in order to translate these results into clinical practice. In conclusion, AgNP-2 is a promising candidate for combatting Candida biofilms, offering a nanotechnology-based strategy to address the growing challenge of antifungal resistance in mucosal and device-associated infections.
